# Gene Delivery Potential of Biofunctional Carbonate Apatite Nanoparticles in Lungs

**DOI:** 10.1155/2014/646787

**Published:** 2014-07-21

**Authors:** Suleiman Yusuf Alhaji, Ezharul Houque Chowdhury, Rozita Rosli, Fatma Hassan, Syahril Abdullah

**Affiliations:** ^1^Medical Genetics Laboratory, Clinical Genetics Unit, Faculty of Medicine and Health Sciences, UPM, 43400 Serdang, Selangor, Malaysia; ^2^Jeffry Cheah School of Medicine and Health Sciences, Monash University Malaysia, 46150 Bandar Sunway, Selangor, Malaysia; ^3^UPM-MAKNA Cancer Research Laboratory, Institute of Bioscience, UPM, 43400 Serdang, Selangor, Malaysia; ^4^Genetics & Regenerative Medicine Research Centre, Faculty of Medicine & Health Sciences, Universiti Putra Malaysia, UPM, 43400 Serdang, Selangor, Malaysia; ^5^Molecular Pathology Research Group, Faculty of Pharmacy and Biotechnology, German University, New Cairo 11835, Egypt

## Abstract

Existing nonviral gene delivery systems to lungs are inefficient and associated with dose limiting toxicity in mammalian cells. Therefore, carbonate apatite (CO_3_Ap) nanoparticles were examined as an alternative strategy for effective gene delivery to the lungs. This study aimed to (1) assess the gene delivery efficiency of CO_3_Ap *in vitro* and in mouse lungs, (2) evaluate the cytotoxicity effect of CO_3_Ap/pDNA *in vitro*, and (3) characterize the CO_3_Ap/pDNA complex formulations. A significantly high level of reporter gene expression was detected from the lung cell line transfected with CO_3_Ap/pDNA complex prepared in both serum and serum-free medium. Cytotoxicity analysis revealed that the percentage of the viable cells treated with CO_3_Ap to be almost similar to the untreated cells. Characterization analyses showed that the CO_3_Ap/pDNA complexes are in a nanometer range with aggregated spherical structures and tended to be more negatively charged. In the lung of mice, highest level of transgene expression was observed when CO_3_Ap (8 *μ*L) was complexed with 40 *μ*g of pDNA at day 1 after administration. Although massive reduction of gene expression was seen beyond day 1 post administration, the level of expression remained significant throughout the study period.

## 1. Introduction

Current understanding of genetic approaches in the treatment of lung genetic diseases reveals that enhancing the transportation of gene into the airway cells is a critical step for improving lung gene therapy [1]. The physical barriers to the airway gene transfer and enzymatic activities in the lung that may render the gene transfer ineffective have to be overcome. Although widely used, cationic lipids are generally found to be ineffective in gene delivery to the mammalian cells [2], especially in the presence of serum [3]. In addition, only a few have impressive activity* in vivo* [4]. Alteration of lipoplex structure in term of size, surface charge, and lipid composition occurs when it is being exposed to a large amount of DNA, proteins, and/or polysaccharides in blood, mucus, epithelial lining, or even tissue matrix [5]. In some cases, pulmonary hypotension, induction of inflammatory cytokines, and tissue infiltration of neutrophils have been reported following aerosolisation of lipid formulations into the lungs [6].

Lately, nonviral gene delivery system in the form of nanoparticle has emerged as an exciting alternative to the conventional nonviral systems for gene therapy of many genetic diseases [7]. Dextran spermine (D-SPM) nanoparticles were found to facilitate gene delivery and subsequent protein expression in the mouse lung [8]. The expression efficiency from D-SPM/pDNA was mostly dependent on the weight-mixing ratio of D-SPM to pDNA, amount of pDNA used, and time-point after administration. Unfortunately, encapsulated pDNA protection conferred by the nanoparticle against degradation by nucleases was minimal and its safety profile in lungs is questionable [9]. To this end, nanoparticles in form of polymers have been regarded as the new promising DNA carrier for pulmonary system [10]. Gold nanoparticles prepared by combining three different leaf powders of* T. nucifera*,* C. japonicum*, and* N. indicum* were also evaluated on 3T3-L1 cell line. Particles absorption was remarkable but the cells exhibited some dose dependent cytotoxicity [11].

It has been reported that nucleic acid complexed with biofunctional carbonate apatite (CO_3_Ap) nanoparticles have remarkable properties capable of mediating high level of gene delivery* in vitro. *These are due to the biodegradability, strong affinity for DNA, and biocompatibility of the CO_3_Ap [12]. Previous studies showed that these particles possess high dissolution rate in endosomal acidic pH, leading to the rapid release of the bound DNA for high levels of protein expression in various cell lines [13]. Moreover, because of their nanosize dimensions and sensitivity to low pH, they can be quickly degraded when taken up by the cells in their acidic vesicles, without any indication of toxicity. These unique properties hold the promise for their applications in the mammalian cells.

Studies on CO_3_Ap nanoparticles mediated gene delivery to HeLa cells demonstrated significant reporter gene expression compared to lipofection and calcium phosphate coprecipitation [13]. Outstanding expression efficiency was observed in NIH 3T3 cells, which resulted in over 50-times higher expression when compared to the existing conventional methods. Transgene expression was also significantly higher in mouse primary hepatocytes [14]. Efficient reporter gene expression from pEGFP-N2 in embryonic teratocarcinoma stem cells utilizing CO_3_Ap nanoparticles as the gene carrier system has been reported [15]. The study also suggested that the addition of specific membrane protein such as fibronectin to the surface of CO_3_Ap complex could enhance the delivery and consequently improved gene expression in the cells. In addition,* in vivo* gene delivery efficiency of the modified carrier system was evaluated using mice bearing A549 tumors [16]. These developments provide an exciting hope for the use of the CO_3_Ap gene delivery system in stem-cell-based therapy.

Although CO_3_Ap nanoparticles have been proven to be effective in the delivery of genes into various cell types* in vitro*, no study has been performed to elucidate their efficiency in the lung to date. Here, we assessed the transfection efficiency of the CO_3_Ap nanoparticles/plasmid DNA (pDNA) complex in the lung cell line and mouse lungs. The* in vitro *analysis was performed to properly understand the gene delivery, expression efficiency, toxicity profiles, and the physical characteristics of the complex formulations. The subsequent study assessed the most optimal conditions for gene expression in the mouse lungs. It was hypothesized that CO_3_Ap is an efficient gene delivery system to the lung cell lines and can be subsequently used for gene transfer to the mouse lung.

## 2. Material and Methods

### 2.1. CO_3_Ap/pDNA Complex Formulations for Transfection

To generate the CO_3_Ap particles, 1 mL of serum-free 44 mM HCO_3_^−^-buffered DMEM (Gibco BRL, CA, USA) (pH 7.4) containing 0.905 mM NaH_2_PO_4_ *·*2H_2_O solution with 24 mM D-glucose, 54 mM NaCl, was mixed with 1 to 12 *μ*L of 1 M CaCl_2_ solution (Calbiochem, Japan) in a 1.5 mL centrifuge tube prior to incubation at 37°C for 30 min. For simplification, the CO_3_Ap particle prepared with 1 to 12 *μ*L of CaCl_2_ solution will be described as CO_3_Ap (1 *μ*L) to CO_3_Ap (12 *μ*L) hereafter. To produce CO_3_Ap/pDNA complex formulation, 2 *μ*g of plasmid DNA (pDNA) pCIKLux (kindly donated by the Gene Medicine Research Group, University of Oxford) with firefly luciferase gene was immediately added to the particle preparation medium following the addition of CaCl_2_, with incubation at 37°C for 30 min. The complex formulations were finally made by the addition of 10% fetal bovine serum (FBS) (Gibco BRL, USA). Another set of CO_3_Ap/pDNA complex was prepared with FBS excluded at the final stage of the formulation protocol.

### 2.2. *In Vitro* Transfection

Human nonsmall carcinoma lung cell line (H1299) was seeded at a seeding density of 50,000 cells in 24 well plates a day before transfection. On the day of transfection, the growth medium was removed and replaced with various formulations of CO_3_Ap/pDNA complexes. After 4 hr, the transfection medium was replaced with 1 mL serum medium and the cells were incubated for 48 hr.

Branched 25 kDa PEI (Sigma-Aldrich, MO, USA) was used as a positive control. The PEI/pDNA was prepared at 10 : 1 N : P ratio, with 9 *μ*g of pDNA. The total of 420 *μ*L of the mixture complexes in Opti-MEM (Invitrogen) was added to each well and incubated for 24 hr. The media were then replaced with fresh complete media and the cells were further incubated for another 24 hr.

### 2.3. Cytotoxicity Study of the CO_3_Ap/pDNA Complex Formulations on the Cells

3-(4,5-Dimethylthiazol-2-yl)-2,5-diphenyl tetrazolium bromide (MTT) 5 mg/mL was used to evaluate the level of cytotoxicity of CO_3_Ap/pDNA complex formulations on H1299 cell line. Prior to the analysis, the cells were seeded at a seeding density of 1.0 × 10^4^ cells in 96-well plate with serum supplemented DMEM medium and incubated for 20 hr. The growth medium was replaced with either CO_3_Ap (4 *μ*L)/pDNA, CO_3_Ap (5 *μ*L)/pDNA, CO_3_Ap (6 *μ*L)/pDNA, or CO_3_Ap (7 *μ*L)/pDNA complexes. After 4 hr of incubation, the complex formulation medium was replaced with a complete growth medium. Following 16 hr of incubation, 20 *μ*L of MTT was added to each well. The cells were then incubated for another 4 hr. Then, 100 *μ*L of dimethyl sulfoxide (DMSO) (Sigma-Aldrich, MO, USA) was added to the MTT treated cells. PEI/pDNA was used as a control. Cell viability was observed using an ELISA reader (ASYS Hitech, GmbH, Austria) at a wavelength of 570 nm and analyzed by a MikroWin 2000 software (ASYS Hitech, GmbH, Austria). Wavelength of 690 nm was used as a reference filter. Untreated cells were taken as control with 100% viability. The (%) of relative cell viability was compared to control cells and was calculated by [absorbance]_sample_/[absorbance]_control_ × 100%.

### 2.4. Size Determination of CO_3_Ap/pDNA Complex

The mean particle size of CO_3_Ap/pDNA formulations was measured using a size analyzer (Nanophox, Sympatec, Germany). Complex formulations of CO_3_Ap (4 *μ*L)/pDNA, CO_3_Ap (5 *μ*L)/pDNA, CO_3_Ap (6 *μ*L)/pDNA, and CO_3_Ap (7 *μ*L)/pDNA with 2 *μ*g of pDNA prepared in medium with 10% of FBS were prepared. The formulations were then dispersed in 2 mL nuclease-free water, making a total volume of 3 mL of particle suspension. Each sample was immediately loaded in an UV-Transparent Spectrophotometry Cuvettes (BrandTech Scientific, USA). Size determinations were performed at 25°C in triplicates in unweighted sample analysis.

### 2.5. Morphological Analysis of CO_3_Ap/pDNA

Freshly prepared CO_3_Ap/pDNA complexes in medium with serum that showed evidence of significant gene expression in the lung cell line were prepared. Pelleted complexes were dissolved in 1 mL nuclease-free water, centrifuged at 14000 rpm for 10 min at 4°C, and then it dispersed in 900 of  *μ*L nuclease-free water. Fifty *μ*L of the suspended pellets was placed on an SEM stage and left to dry at 50°C for 10 min. The morphological appearance of the complexes was observed by a high resolution field emission scanning electron microscope (FESEM) (Jeol JSM-7600F, Japan).

### 2.6. CO_3_Ap/pDNA Surface Charges Determination

Complexes of CO_3_Ap (4 *μ*L)/pDNA to CO_3_Ap (9 *μ*L)/pDNA were prepared with 2 *μ*g of pDNA in 1 mL of freshly prepared serum-free DMEM (Gibco BRL, USA). After incubation for 30 min at 37°C, 10% FBS was added to the respective complexes. The mixtures were pipetted into UV-Transparent Spectrophotometry Cuvettes (BrandTech Scientific, USA). Relative charge intensity values were obtained by Zeta-Sizer (Malvern, Germany) and the results are presented as mean value of the resultant charges obtained.

### 2.7. Comparative Gel Retardation Assay

CO_3_Ap/pDNA retardation capacity following agarose gel electrophoresis in comparison to PEI/pDNA and Lipofectamine/pDNA was assessed. CO_3_Ap (8 *μ*L) was mixed with 3 *μ*g of pCIKLux in 100 *μ*L serum-free DMEM. The Lipofectamine/pDNA complex was prepared at a ratio of 5 : 1 following the protocol suggested by the manufacturer (Invitrogen, USA). PEI/pDNA complex was prepared as described earlier. The degree of complex retardation was determined on 0.8% agarose gel electrophoresis at 75 V for 30 min. The DNA band was visualized under Gel documentation system (G:BOX BioImaging System) (Syngene, USA) and the image was analyzed using a Genesnap software (Syngene, USA).

### 2.8. DNase I Protection Assay

Complexes of CO_3_Ap (3 *μ*L)/pDNA to CO_3_Ap (8 *μ*L)/pDNA were prepared with 2 *μ*g of pDNA in 1 mL freshly prepared complex solution. The complexes were incubated with 1 Unit of DNase I (Thermo scientific, Fermentas, USA) at 37°C for 3 min in a final volume of 10 *μ*L, as suggested by the manufacturer. The digestion was halted by the addition of stop solution. The integrity of pDNA released from the complex was assessed in 0.8% agarose gel electrophoresis. The degree of pDNA protection by CO_3_Ap encapsulation was compared with the naked pDNA in complex solution and PEI/pDNA complex formulation.

### 2.9. Animals

BALB/c mice were maintained in individually ventilated cages (IVC) (Rair Isosystem, Laboratory Product Inc., USA) and fed with standard chow and water* ad libitum*. The mice were allowed to acclimatize for at least 7 days prior to the experiment. Approval for the experimental procedure was obtained from the Animal Care and Use Committee (ACUC) of the Faculty of Medicine and Health Sciences, Universiti Putra Malaysia, with the approval number UPM/FPSK/PADS/BR-UUH/00427. All the experiments were carried out in accordance to the guidelines for animal experimentations of Universiti Putra Malaysia.

### 2.10. *In Vivo* Gene Delivery

General preparation of CO_3_Ap/pDNA complexes for* in vitro* experimentation has been described previously. However, in this study, the CO_3_Ap particles were made with 6 *μ*L to 10 *μ*L of 1 M CaCl_2_ and complexed with 10 *μ*g of endotoxin free pCIKLux, to a total dosing volume of 100 *μ*L. As for the increasing pDNA study, the amount of pDNA was sequentially increased from 10 to 100 *μ*g, with constant amount of CO_3_Ap made from 8 *μ*L CaCl_2_, in an approximate volume of 100 *μ*L serum-free medium. For the control group, PEI/pDNA was prepared as previously described, with the modification of 20 *μ*g of pDNA used, to a total volume of 100 *μ*L in a prewarmed Opti-MEM (Gibco, USA). The complexes were incubated at room temperature for 20 min.

Female BALB/c mice (6–8 weeks old) were anesthetized in a fume hood using 100% isoflurane (Nicholas Piramal (I), Ltd., UK) inhalation until a balanced state of anaesthesia was achieved. One hundred *μ*L of the complexes was administered into the mouse lung via the nasal route. While the untreated group was not subjected to any treatment, another group of mice was instilled with 10 *μ*g of naked pDNA in 100 *μ*L of buffered DMEM solution. Six mice per group (*n* = 6) were used to assess the gene delivery potential of CO_3_Ap to the mouse lung. The mice were sacrificed 48 hr after administration (unless stated otherwise) by cervical dislocation and their trachea and lungs were harvested and immersed in 200 *μ*L of 1x reporter lysis buffer (Promega, WI, USA). The samples were stored in a −80°C freezer. After thawing, tissues were homogenized using Ultra-Turrax (IKA, Staufen, Germany) and the lysates were then passed through a QIAshredder column (Qiagen).

### 2.11. Reporter Gene Activity

The luciferase activity for both* in vitro* and* in vivo* samples was measured using a luciferase assay kit (Promega) on a GloMax 20/20 luminometer (Promega), following the manufacturer's protocol. The relative light units (RLUs) were normalized against protein concentration in the cell extracts, which was quantified using a Bio-Rad DC Protein Assay (Bio-Rad Laboratories, CA, USA). The expression efficiency is presented as mean RLU per milligram of cell protein (RLU/mg protein).

### 2.12. Statistical Analysis

Distribution-free, nonparametric Mann-Whitney *U* test was utilized to compare two unpaired groups of variables, while one-way ANOVA test was applied to compare multiple groups. The error bars on graph data represent standard error of the mean (SEM) for all data sets. Data are presented as value ± SEM and considered to be statistically significant if *P* values are < 0.05. Analysis was performed using SPSS for Windows, Version 17.0.

## 3. Results

### 3.1. Gene Expression Efficiency

CO_3_Ap particles were formulated from 1 to 12 *μ*L of CaCl_2_ (henceforth described as CO_3_Ap (1 *μ*L)–CO_3_Ap (12 *μ*L) for simplification) and then mixed with 2 *μ*g of pDNA to produce various formulations of CO_3_Ap/pDNA. The complexes were prepared in medium supplemented with or without serum.

**Figure 1:**
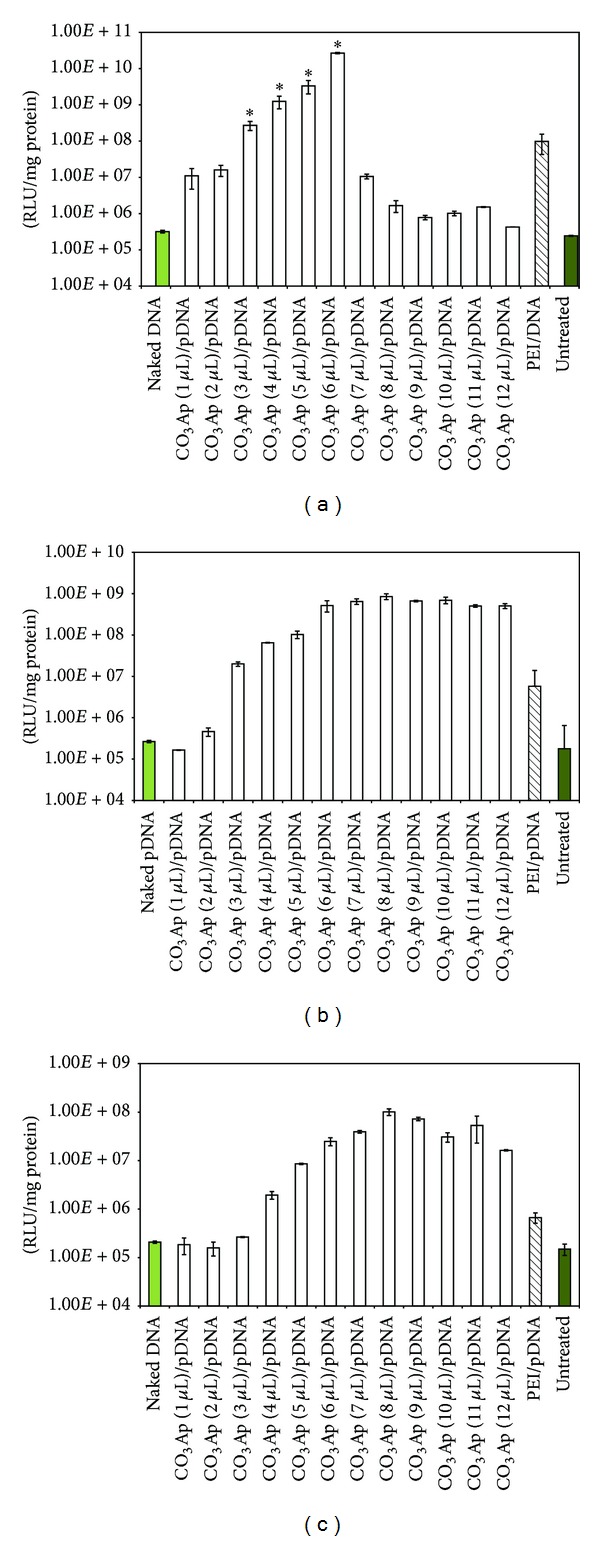
Lung cells were transfected with various formulations of CO_3_Ap/pDNA, carrying constant amount of pDNA (2 *μ*g). (a) Transfection efficiency of CO_3_Ap/pDNA complexes prepared in 1 mL DMEM supplemented with 10% serum protein. (b) Transfection efficiency of CO_3_Ap/pDNA complexes prepared in 1 mL serum-free DMEM. (c) Transfection efficiency of CO_3_Ap/pDNA complexes prepared in 100 *μ*L serum-free DMEM. Luciferase expression was measured as RLU/mg protein at 48 hr after transfection. The error bar represents standard error of the mean (SEM) for all data sets. The result is considered to be statistically significant if *P* values were <0.05. Data are presented as mean ± S.E.M of experiments conducted in triplicate.

H1299 was successfully transfected with the various formulations of CO_3_Ap/pDNA. Luciferase expression was observed to increase from CO_3_Ap (1 *μ*L)/pDNA and reached significantly high at CO_3_Ap (4 *μ*L)/pDNA, CO_3_Ap (5 *μ*L)/pDNA, and CO_3_Ap (6 *μ*L)/pDNA (1.25 × 10^9^, 3.34 × 10^10^ and 2.68 × 10^11^ RLU/mg, resp.) when compared to PEI/pDNA (9.8 × 10^7^ RLU/mg) and the untreated (2.44 × 10^5^ RLU/mg) groups, at 48 hr after transfection (Figure 1(a)). A decline in gene expression was observed at CO_3_Ap (12 *μ*L)/pDNA. To investigate the role of serum protein in particle formation, we performed transfection using a test group in which the serum protein was excluded in the complex formulation. The results showed evidence of overall decline in gene expression to about 30% RLU/mg protein (Figure 1(b)) compared to transfections using a CO_3_Ap complex formulation in medium with serum (Figure 1(a)). However, the difference was not significant.

In the following study, the complex was prepared in 100 *μ*L medium without serum and then topped up to 1 mL with DMEM without serum for transfection purposes. The transfection medium was replaced with DMEM supplemented with 10% serum 4 hr after transfection. Reporter gene expression was observed 48 hr after transfection and the result showed that the reporter gene expression was significantly higher than the control groups when 8 *μ*L of 1 M CaCl_2_ was used to prepare the CO_3_Ap [CO_3_Ap (8 *μ*L)/pDNA] (Figure 1(c)). Nevertheless, the results also showed an overall decrease in gene expression compared to transfection using 1 mL of the complex media in both serum (Figure 1(a)) and without serum (Figure 1(b)).

### 3.2. Cytotoxicity Analysis

**Figure 2:**
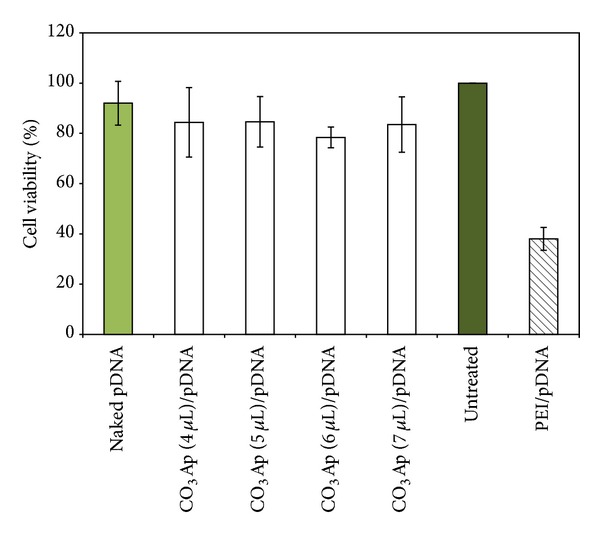
MTT Assay on CO_3_Ap/pDNA transfected cells. Lung cells were transfected with formulations of CO_3_Ap for 4 hr followed by tetrazolium assay. No significant difference between the CO_3_Ap/pDNA treated groups and the untreated group was observed. The error bar represents standard error of the mean (SEM) for all data sets. The result is considered to be statistically significant if *P* values were <0.05. Data are presented as mean ± S.E.M of experiments conducted in triplicate.

Tetrazolium assay was performed to assess the cytotoxicity of the CO_3_Ap/pDNA complex formulation in the lung cells. The relative cell viability percentage was compared to the untreated cells, which were considered as the control with 100% viability. The results showed no significant difference between the CO_3_Ap/pDNA treated groups, with about 80% viable cells, when compared to the untreated group. Mild cytotoxicity was observed following MTT assay on CO_3_Ap/pDNA transfected H1299 (Figure 2). However, the viable cell density in the PEI/pDNA group was significantly lower than the untreated and CO_3_Ap treated groups, with less than 50% viable cells.

### 3.3. Characteristics of CO_3_Ap/pDNA Complexes

**Figure 3:**
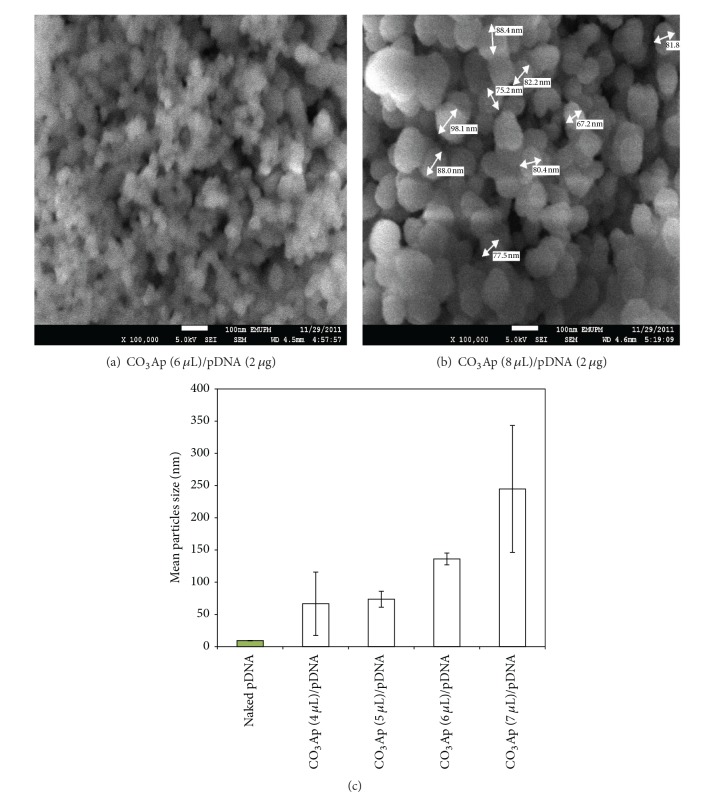
Morphological appearance and size of CO_3_Ap/pDNA complexes. Formulated CO_3_Ap complexes using various amount of CaCl_2_ with 2 *µ*g of pDNA were subjected to FESEM and Nanophox analyses. Complex formulation by (a) CO_3_Ap (6 *μ*L)/pDNA shows much smaller aggregated particles when compared to (b) CO_3_Ap (8 *μ*L)/pDNA with individual particle size of about 150 nm. Scale: (×100 000) (c) CO_3_Ap/pDNA size analysis. The complex size was found to be proportional to the increasing amount of CaCl_2_ used to prepare the CO_3_Ap/pDNA formulations. The error bar represents standard error of the mean (SEM) for all data sets. Data are presented as mean ± S.E.M of experiments conducted in triplicate.

The formulations of CO_3_Ap/pDNA that gave significant transgene expression were prepared and evaluated for their physical characteristics. CO_3_Ap formulated with 4–9 *μ*L CaCl_2_ and complexed with 2 *μ*g pDNA was prepared as described previously. The complexes were subjected to size determination by Nanophox (Sympatec, Germany). The result showed that the particle size distribution in the formulations was not homogenous. CO_3_Ap (4 *μ*L)/pDNA, CO_3_Ap (5 *μ*L)/pDNA, CO_3_Ap (6 *μ*L)/pDNA, and CO_3_Ap (7 *μ*L)/pDNA presented an average size of 66.5 ± 0.2, 73.6 ± 49.1, 136.1 ± 9.1, and 244.8 ± 98.4 nm, respectively, (Figure 3(c)). The size increase was proportional to the increasing amount of CaCl_2_ used to prepare the CO_3_Ap for CO_3_Ap/pDNA complex generation (*P* < 0.05). All of the CO_3_Ap/pDNA complexes were in the nanosize range despite the fact that the size distribution of the formulation solution was not uniform.

Next, the morphology of CO_3_Ap (6 *μ*L)/pDNA and CO_3_Ap (8 *μ*L)/pDNA complexes was analyzed using FESEM. It was observed that the complexes exhibit aggregated spherical shape, which increased in size proportionate to the increasing amount of CaCl_2_ used to prepare the CO_3_Ap particle (Figures 3(a) and 3(b)). CO_3_Ap (6 *μ*L)/pDNA exhibited much smaller particle size than CO_3_Ap (8 *μ*L)/pDNA under the same formulation condition. In both formulations, the particles were all in nanosize with an individual particle complex size of 100–150 nm for CO_3_Ap (6 *μ*L)/pDNA and 300–400 nm for CO_3_Ap (8 *μ*L)/pDNA. The findings are in concordance with the Nanophox readings showing the size of 136.1 ± 9.1 nm for CO_3_Ap (6 *μ*L) and 244.8 ± 98.4 nm for CO_3_Ap (7 *μ*L).

Surface charges of the formulated particles ranging from (CO_3_Ap (4 *μ*L)/pDNA) to (CO_3_Ap (9 *μ*L)/pDNA) were observed using Zeta Sizer. The Zeta potentials measurement of the resulting complexes revealed that the particles possessed slightly negative surface charge (Table 1). The table also shows that as the particles size increases (by increasing the volume of CaCl_2_ used to prepare CO_3_Ap) the surface charge density also decreases.

### 3.4. Gel Retardation and DNase Protection Assays

**Figure 4:**
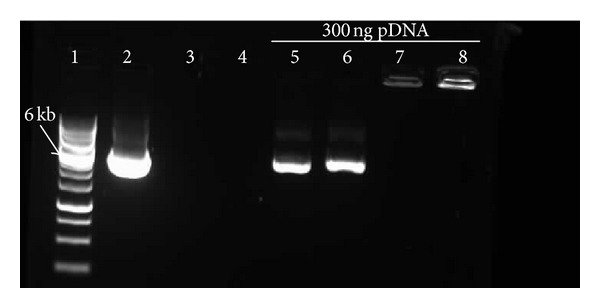
Comparative retardation assay of CO_3_Ap/pDNA, Lipofectamine/pDNA, and PEI/pDNA. Lane1: 1 kb DNA ladder, Lane 2: 1 *μ*g uncut pDNA, Lane 3: DMEM, Lane 4: CO_3_Ap, Lane 5: pDNA in DMEM solution, Lane 6: CO_3_Ap (8 *μ*L)/pDNA; Lane 7: Lipofectamine/pDNA; and Lane 8: PEI/pDNA. The respective formulations were run on 0.8% agarose gel for 45 min at 75 V.

Here, the surface charges condensation behavior of CO_3_Ap (8 *μ*L)/pDNA was compared with the conventional carrier systems (25 kDa PEI solution and Lipofectamine 2000 complexes) through their retardation capacity in agarose gel electrophoresis. CO_3_Ap (8 *μ*L)/pDNA showed a clear band within the agarose gel whereas lipofectamine and PEI showed band in the well, implying greater retardation capacity compared to the CO_3_Ap (8 *μ*L)/pDNA (Figure 4). No difference in the band intensity between the pDNA encapsulated CO_3_Ap (8 *μ*L)/pDNA and the linearized pDNA was observed.

Next, an assessment of the protection level offered by CO_3_Ap to the encapsulated pDNA at various formulations was performed. CO_3_Ap (4 *μ*L)/pDNA to CO_3_Ap (8 *μ*L)/pDNA formulations encapsulating 2 *μ*g of pDNA were subjected to DNase I treatment. Figure 4 shows that the CO_3_Ap/pDNA was able to migrate within the agarose gel, hence, it was expected to see a band of pDNA in the gel after DNase I treatment if the pDNA was effectively protected by the CO_3_Ap encapsulation. In this current study, the presence of a clear band of approximate molecular weight of 5632 bp in the gel indicated that the pDNA was preserved from being degraded.

**Figure 5:**
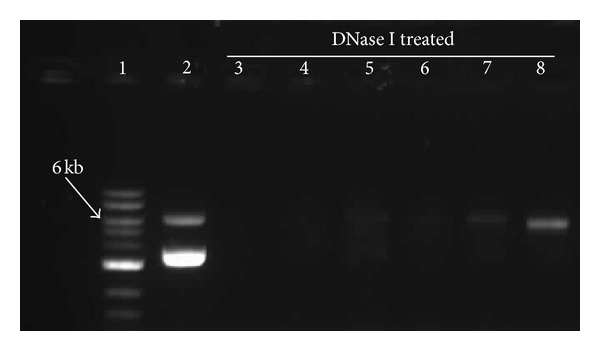
Agarose gel electrophoresis of CO_3_Ap/pDNA complexes at different concentrations of CO_3_Ap following DNase I treatment. Lane 1: 1 kb DNA ladder; Lane 2: 1 *µ*g super coiled pDNA; Lane 3: Naked pDNA; Lane 4: CO_3_Ap (4 *µ*L)/pDNA; Lane 5: CO_3_Ap (5 *µ*L)/pDNA; Lane 6: CO_3_Ap (6*µ*L)/pDNA; Lane 7: CO_3_Ap (7 *µ*L)/pDNA; and Lane 8: CO_3_Ap (8 *µ*L)/pDNA. All the formulations were prepared in serum-free media and the respective formulations were run on 0.8% agarose gel for 45 min at 75 V.

Clear bands were observed in the lane containing pDNA without the DNase treatment, indicating the presence of the pDNA (Figure 5). The absence of pDNA band in the DNase I treated CO_3_Ap (4 *μ*L)/pDNA and CO_3_Ap (5 *μ*L)/pDNA lanes showed that the pDNA was completely degraded by the DNase I enzyme. A faint band of pDNA was seen in the DNase I treated CO_3_Ap (6 *μ*L)/pDNA and CO_3_Ap (7 *μ*L)/pDNA lanes compared to CO_3_Ap (4 *μ*L)/pDNA and CO_3_Ap (5 *μ*L)/pDNA DNase I treated groups. This shows that CO_3_Ap (6 *μ*L) and CO_3_Ap (7 *μ*L) offered minimal pDNA protection from the effect of DNase I. A defined band was observed in the CO_3_Ap (8 *μ*L)/pDNA lane, indicating that the DNA was sufficiently protected against degradation. Unprotected free pDNA in DNase treated group was completely digested by DNase I treatment, hence no pDNA band was observed in the gel documentation system.

### 3.5. *In Vivo* Gene Delivery Analysis

**Figure 6:**
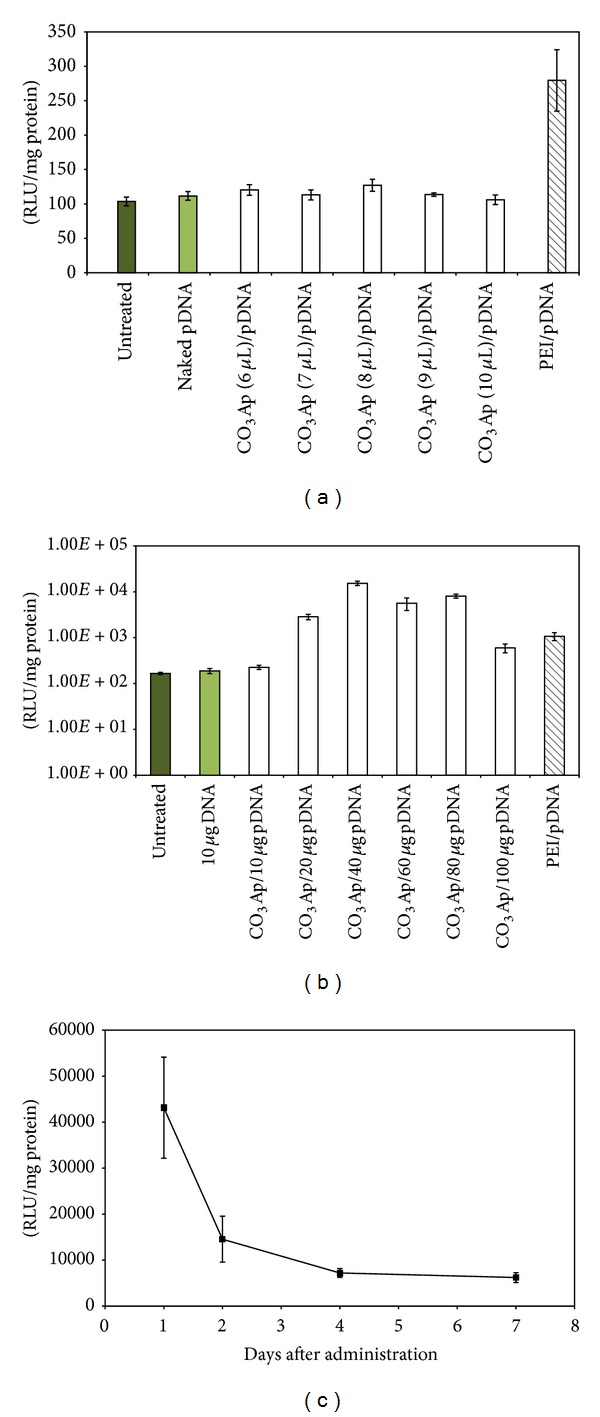
BALB/c mice lung and trachea luciferase reporter gene expression analysis. (a) Gene delivery efficiency of various formulations of CO_3_Ap with constant amount of pDNA (10 *μ*g). (b) Increasing pDNA study using 10–100 *μ*g of pDNA with constant amount of CO_3_Ap (8 *μ*l). (c) Time point experiment utilizing 40 *μ*g of pDNA with constant amount CO_3_Ap (8 *μ*l). Luciferase expression was measured as RLU/mg protein. The error bar represents standard error of the mean (SEM) for all data sets. The result is considered to be statistically significant if *P* values were <0.05. Data are presented as mean ± S.E.M from 6 animals.

As demonstrated in the* in vitro* studies, the level of transgene expression mediated by CO_3_Ap/pDNA complex formulations was promising in the lung cell lines. The cytotoxicity analysis also revealed that the formulations were considerably nontoxic. Therefore, we further investigated the gene delivery potential of the complex in the lung of BALB/c mice. Surprisingly, the reporter gene expression levels in mouse lungs from CO_3_Ap (6 *μ*L)/pDNA to CO_3_Ap (8 *μ*L)/pDNA complexes, formulated with 10 *μ*g of pDNA, at 48 hr after delivery were not impressive (Figure 6(a)). CO_3_Ap (8 *μ*L)/pDNA showed a trend of higher gene expression compared to other CO_3_Ap treated groups with an average RLU/mg protein of 127.04 ± 5.72, but the increase was not statistically significant. The levels of reporter gene expression from mice treated with all formulations of CO_3_Ap/pDNA were substantially inferior to PEI/pDNA treated group (279.4 ± 30.96 RLU/mg protein). In addition, the reporter gene expression from the CO_3_Ap/pDNA treated experimental groups did not present significant difference when compared to the untreated and naked pDNA treated groups.

Albeit not being statistically significant, 10 *μ*g of the pDNA complexed in CO_3_Ap (8 *μ*L)/pDNA was able to present a trend of higher reporter gene expression at 48 hr after administration in mouse lungs. Therefore, we performed another experiment by increasing the amount of the pDNA up to 100 *μ*g, in 20 *μ*g increment, in the CO_3_Ap (8 *μ*L)/pDNA complex formulation. The purpose of this study was to determine the optimal amount of pDNA that can be encapsulated in CO_3_Ap (8 *μ*L)/pDNA formulation to present significant gene expression in the mouse lung. The result in Figure 6(b) shows that there was a remarkable increase in the reporter gene expression in the mouse lungs following the increasing amount of the pDNA used in the formulation. Significantly high levels of gene expression were observed from CO_3_Ap (8 *μ*L)/pDNA complexed with 20 *μ*g to 80 *μ*g of pDNA with RLU/mg value from 8078.0 ± 5.5 × 10^2^ when compared to PEI/pDNA treated group (1.07 × 10^3^ ± 1.49 × 10^2^ RLU/mg) and untreated group (66.1 ± 7.5 RLU/mg) (*P* < 0.05). However, increasing the amount of the pDNA to 100 *μ*g in CO_3_Ap (8 *μ*L)/pDNA did not improve the level of reporter gene expression.

Time point experiment was performed to determine the period when the level of luciferase gene expression was at its highest in the mouse lung. Mice were instilled with CO_3_Ap (8 *μ*L)/pDNA complex formulation, with the amount of pDNA kept at 40 *μ*g. The mice were sacrificed and the lungs and trachea were harvested for luciferase gene expression analysis at 1, 2, 4, and 7 days after administration. The result showed that the luciferase gene expression peaked at day 1 after administration, with significant luciferase activity of 4.3 × 10^4^ ± 1.09 × 10^3^ RLU/mg when compared to the other time points (Day 2: 1.4 × 10^4^ ± 4.99 × 10^3^ RLU/mg, Day 4: 7.20 × 10^3^ ± 9.4 × 10^2^ RLU/mg, and Day 7: 6.2 × 10^3^ ± 1.0 × 10^3^ RLU/mg) and to the untreated and naked pDNA treated groups (51.3 ± 13.5 RLU/mg and 49.0 ± 5.5 RLU/mg, resp.) (*P* < 0.05) (Figure 6(c)). A remarkable decline in the luciferase activity was observed from day 2 onwards. However, significantly higher gene expression was observed at all time points analyzed when compared to the control groups.

## 4. Discussion

Nucleic acid delivery to lung cell lines and in animal model by nonviral approach for gene expression analysis has been challenging. A nonsmall cell lung carcinoma cell line (H1299) and BALB/c mice as the animal model were utilized in this study. Inbred BALB/c mouse strain was employed because it is more receptive to lung gene delivery when compared to other mouse model, such as the SCID mouse [17]. Therefore, it serves as a useful tool for assessment of gene delivery and also a model of choice for investigating transgene expression for future lung gene therapy.

Successful gene delivery and expression were obtained following transfection of H1299 cell lines with CO_3_Ap/pDNA complex formulations with or without serum. However, a trend of overall decline in gene expression was observed when the cells were transfected with the CO_3_Ap/pDNA formulations without serum protein when compared to formulations with serum protein. There could be two reasons for this observation. One, it has been reported earlier that CO_3_Ap particle prepared in serum protein resulted in the formation of microscopically visible particles that moved in a brownian fashion in the solution [18]. This finding suggests that serum adsorption on the particles complex surface could facilitate effective interaction of the particles with cell surface membrane, thereby enhancing cellular uptake of the complex formulation and hence increases gene expression. Second, the presence of serum protein may regulate particles formation, perhaps in regulating the particle size or in the stability of the particle, which are known to play a major role in gene delivery efficiency since large particles are phagocytosed less efficiently than the small particles [19].

As demonstrated in the* in vitro* transfection result, 1–3 *μ*L of CaCl_2_ in the CO_3_Ap formulations in both serum (Figure 1(a)) and serum-free medium (Figure 1(b)) did not present significant gene expression when compared to the controls. This could be explained by the fact that negligible amount of particles was formed, which was insufficient to mediate gene delivery. However, significant gene expression was observed when higher volume of CaCl_2_ was used in the CO_3_Ap formulation complex prepared with serum (Figure 1(a)) or without serum (Figure 1(b)). In general, this study has shown that the amount of CaCl_2_ used to formulate the particles is important in determining the efficacy of CO_3_Ap as a gene delivery vector. A similar observation was reported by Chowdhury and Akaike (2007), where the CO_3_Ap particle formation is solemnly dependent on the addition of the optimal volume of CaCl_2_ solution [13]. To mimic the optimal amount of CO_3_Ap/pDNA a mouse lung can accommodate for subsequent* in vivo* study, we formulated the complex formulation in 100 *μ*L amount of solution and then topped up to the required amount for transfection purpose. Reporter gene expression was found to be significantly higher than the controls, as the amount of CaCl_2_ used in the formulation increased (Figure 1(c)). Similar to the results shown in Figures 1(a) and 1(b), the gene expression did not improve after the optimal amount of CaCl_2_ was used to prepare the particles. However, the overall gene expression levels in this study were not as impressive as that of earlier findings, with highest RLU/mg value recorded at 10^8^. In contrast to the result obtained in the 100 *μ*L formulation, transfection efficiency was comparatively high when the cells were transfected with the formulations prepared with or without serum in 1 mL transfection medium. This may indicate that the particle complexes were quite numerous and more aggregated in reduced volume and subsequently affects gene delivery efficiency.

The optimal formulations of CO_3_Ap that showed evidence of significant gene expression were used for the* in vitro* cytotoxicity analysis. CO_3_Ap/pDNA delivery into the lung cells indicated that the complex was less toxic (Figure 2). Viable cell densities in all the CO_3_Ap treated groups were 90% when compared to the untreated cell with 100% viability, with no statistical difference. Increasing concentration of CaCl_2_ in the complex formulation could lead to the formation of aggregates (Figures 3(a) and 1(b)). However, it appears that the aggregations of the CO_3_Ap particles did not have a significant effect on the cell viability. CO_3_Ap is one of the components of body hard tissues such as bone and teeth, with remarkable biodegradability [12]. Due to this feature, the particles can be easily excreted out from the cells without compromising the viability of the cells tested. In contrast, the viability of PEI/pDNA treated cells was significantly lower than the untreated group with less than 50% viable cells. PEI was reported to exhibit impressive gene delivery property but causes significant cell death [20], possibly due to the aggregation of mass cationic polymer on the outer cell membrane [21]. This impairs the vital function of the cell's membrane by affecting the cytoskeletal structures which eventually leads to the induction of membrane damage and necrosis of the cells [22].

Glycoprotein, being one of the major components of the serous mucus, may cause aggregation of CO_3_Ap and consequently affects gene delivery efficiency. To test this phenomenon, we replicated the situation* in vitro* by generating the particles and performing transfection in medium with serum. The formulated CO_3_Ap nanoparticle complexes showed numerous particles of different sizes, with overall spherical or grape-like features with smooth surface in an aggregated form on field emission scanning electron microscope (FESEM) (Figures 3(a)-3(b)). Zink formulated nanoparticles also exhibited similar structure under FESEM [23], but with much smaller size compared to CO_3_Ap. The aggregates were quite numerous and increased as the amount of CaCl_2_ used for particle formulation was increased. Although particle aggregations occurred, they did not affect the efficiency in gene delivery. However, particle formulation using CaCl_2_ of more than 6 *μ*L might form relatively larger particles in serum which were difficult to be phagocytosed than the smaller particles [24]. This might lead to the lower gene expression observed.

In general, the results showed that the aggregation and the particle size increase with the corresponding increase of CaCl_2_ used in the complex formulations. This agrees with the earlier findings that a higher number of particles with corresponding size increments were observed when excess CaCl_2_ was added in the complex formulation [13]. The particles formed aggregates with the serum protein but did not affect transfection efficiency [18]. It has been suggested that* in vitro* transfection by gene delivery agent should not be performed in medium with serum as this may impede the rapture and release of the pDNA in the cytosol of the cell which eventually reduces gene expression [25]. However, this study showed that promising gene expression* in vitro* from CO_3_Ap/pDNA was obtained even when the transfection was performed in medium with serum. This implies that the formulations can be used for effective gene delivery in the settings with existing serum protein such as in mouse lungs.

Correlating to the transfection results in Figures 1(a)–1(c), it was shown that negligible gene expression was obtained when 1–3 *μ*L of CaCl_2_ was used to prepare the complex. This could probably be due to the insufficient amount of particles generated that was not adequate to form stable complexes for effective transfection. Highest gene expression was detected from CO_3_Ap formulations prepared with 6 *μ*L CaCl_2_ for transfection in medium with serum (Figure 1(a)) and 8 *μ*L CaCl_2_ for transfection in serum-free medium (Figures 1(b) and 1(c)). It is speculated that the particle size was relatively sufficient to generate stable particle complexes efficient for transfection of lung cells. Particles with small size, which are estimated around 250 nm in this study, have a greater number of molecules on their surface rather than inside [17], thereby creating a large surface area to volume ratio [18]. This will eventually enable mass uptake of the particles into the cells since large surface area provides an avenue for increase in dissolution velocity. In addition, studies have shown that pulmonary epithelial cells are able to uptake materials at a particle size range of 500 nm 10 times more than 1 *μ*m and 100 times more than 2 or 3 *μ*m [19]. The smaller size gene carrier was found to be capable of escaping the clearance effect of the reticuloendothelial system, although it has longer retention time in the system circulation [20].

Gene delivery mediated by the nonviral systems to mammalian cell is mostly facilitated by strong ionic interactions with the cell membrane [26]. It involves mainly electrostatic interactions between the positively charged carrier complexes and the negatively charged cell surface membrane. It was found that all the CO_3_Ap/pDNA complexes possess negatively charged surface. The negative charge (Table 1) and the particle size (Figure 3(c)) increase as the amount of CaCl_2_ used to prepare the CO_3_Ap increased, in the CO_3_Ap/pDNA formulations. It is speculated that the expansion of the particle size is a consequence of the increase in the negative charge. Earlier studies demonstrated that increment of CaCl_2_ in the CO_3_Ap formulation accelerates chemical reaction to promote particle formation. This reduces the ionization and the stability of the complex formulation [12]. Perhaps the molecules repel each other when they are heavily anionic. This may lead to the expansion of the complexes resulting in the increasing size seen in the study. Although the particles possess negative surface charges, they could still mediate gene expression in the lung cells. This is because the particles have a cationic Ca^+^ domain, which electrostatically react with the negative phosphate backbone of the pDNA to form a stable complex for efficient gene delivery into the cell.

Transgene degradation by intracellular nuclease activities is another major concern in lung gene delivery. To mimic the conducting airway system environment having a variety of nuclease activities, CO_3_Ap/pDNA complexes prepared with different concentrations of CaCl_2_ were treated with DNase I. This aimed to evaluate the level of pDNA protection by CO_3_Ap. The protection level conferred by the formulations to the pDNA was minimal, especially when lower concentrations of CaCl_2_ were used to prepare the CO_3_Ap (Figure 4). This is not surprising because lower concentrations of CaCl_2_ may form insufficient amount of particles that can effectively condens the pDNA. This limits the pDNA encapsulation capacity, exposing the pDNA to nuclease's action. Slow mobility of the encapsulated pDNA in the cytoplasm can make it susceptible to the Ca^2+^ sensitive cytoplasmic nucleases. This can restrict the half-life of pDNA in the cytoplasm [27]. However, here we have shown that the CO_3_Ap particles under the right conditions were able to protect the pDNA from the activity of nucleases. This should provide chances of longer retention period and allows mass uptake of the pDNA by the cells.

Effective encapsulation property of pDNA by gene carrier systems is another important requirement for a successful nonviral carrier system. Cellular uptake of pDNA into the cell in the form of vesicles is strongly susceptible to lysosomal degradation, if the pDNA is not being encapsulated efficiently by the gene carrier system. Therefore, the assessment of the encapsulation efficiency of pDNA by CO_3_Ap was evaluated using the gel retardation assay. Comparative electrophoretic mobility analysis of the released pDNA on agarose gel electrophoresis revealed that pDNA complexed with CO_3_Ap has a weaker retardation capacity compared to PEI and Lipofectamine, as the CO_3_Ap/pDNA complex ran within the gel while PEI and Lipofectamine did not migrate from the well of the agarose gel (Figure 5).

Clear band of pDNA was observed in the CO_3_Ap/pDNA lane (Figure 5). There are three possibilities for this observation. First, the CO_3_Ap particles might have released the pDNA during the electrophoretic mobility and the band observed was an unbound pDNA. This suggests that there was only a minimal encapsulation of the pDNA by the CO_3_Ap. Although it may not provide effective protection against lysosomal degradation, minimal encapsulation may be beneficial to the gene delivery system, as it requires little energy for effective release of the pDNA in the cytosol. Second, molecules move through the agarose gel matrix at different rates, which is greatly influenced by the mass to charge ratio. Therefore, the CO_3_Ap particle, which is in nanosize (Figure 2), migrates throughout the matrix pores of the agarose gel easily compared to a microsized particle. More importantly, the surface charge of the CO_3_Ap/DNA could be another factor that can make it possible for the complex formulation to migrate within the gel. The ability of molecules to move within the gel is highly dependent on their surface charges. In the particle surface charge analysis, it has been revealed that the CO_3_Ap/pDNA complexes possessed negative surface charges and the charge density reduced with the increasing amount of CaCl_2_ used in the preparation of the CO_3_Ap particle (Table 1).

Although CO_3_Ap shows minimal encapsulation of pDNA (Figure 4), the CO_3_Ap formulations tested in this study could still adequately encapsulate the pDNA for effective transfection. If the pDNA was not encapsulated, the CO_3_Ap/pDNA groups in the* in vitro* transfection studies would have generated similar gene expression values as the naked pDNA group (Figures 1(a)–1(c)). However, the CO_3_Ap/pDNA groups showed significantly higher gene expression values compared to the naked pDNA group. In addition, the pDNA in the CO_3_Ap/pDNA group was preserved in a supercoiled form when subjected to DNase I. This serves as an added advantage as supercoiled plasmid performs better in transfection since this form of pDNA can reach the perinuclear region more efficiently [28].

The assessment for the most optimal conditions for gene delivery to the mouse lungs was performed, following the successful* in vitro* study. The first* in vivo* study evaluated the amount of CaCl_2_ used in CO_3_Ap/pDNA complex formulations that would give the highest gene expression. CO_3_Ap/pDNA prepared with 6 to 10 *μ*L CaCl_2_ was tested as this range showed promising gene expression in the* in vitro* study. The amount of pDNA was kept to 10 *μ*g as this concentration was shown to be effective in other* in vivo* studies [8]. Unfortunately, insignificant gene expression was observed in all CO_3_Ap/pDNA treated groups when compared to the untreated and naked pDNA groups (Figure 6(a)). Nevertheless, a trend of higher gene expression was seen from the CO_3_Ap (8 *μ*L) pDNA treated mice. PEI/pDNA treated mice group, which served as the positive control for gene delivery, showed the highest level of gene expression. However it has to be noted that 20 *μ*g of pDNA was used in the PEI/pDNA formulation as this is the most optimal condition for PEI/pDNA mediated gene delivery to the mouse lung [29].

Studies by Pringle et al. [30] demonstrated successful gene expression from the delivery of 80 *μ*g of pDNA by Genzyme Lipid GL67A to the mouse lung. Since CO_3_Ap nanoparticles have an inherent advantage of submicronic nature, we speculated that it might provide a large surface area for adequate packaging capacity of pDNA. This led us to perform an increasing pDNA study in the formulation complex to determine the most optimal level of pDNA concentration that can generate an optimal level of gene expression in the mouse lung. Plasmid DNA concentrations in the range of 10 to 100 *μ*g were complexed with CO_3_Ap and analyzed in this study. CO_3_Ap prepared with 8 *μ*L of CaCl_2_ [CO_3_Ap (8 *μ*L)] was used as it showed a trend of higher gene expression in the earlier study (Figure 6(b)). Remarkable gene expression was observed following the instillation of CO_3_Ap (8 *μ*L) complexed with 20 to 80 *μ*g when compared to the untreated and naked pDNA delivery groups (Figure 6(b)). Highest value of gene expression was achieved from the mouse group delivered with CO_3_Ap (8 *μ*L) complexed with 40 *μ*g pDNA [CO_3_Ap (8 *μ*L)/40 *μ*g pDNA], even significantly higher than the positive control (PEI/pDNA). However, a massive decline in gene expression was observed when the amount of pDNA in the formulation was increased to 100 *μ*g. It is speculated that this formulation generates excessive amounts of unbound pDNA in the solution that can inhibit the available bound pDNA from cellular entry and release. A time point study was employed to determine the period when the level of gene expression was at its highest, by using CO_3_Ap (8 *μ*L) complexed with 40 *μ*g pDNA formulation. Generally, transgene expression mediated by nonviral gene delivery in mouse lung is transient [17], with significant expression that only lasted for 2 days. By day 7 after delivery, the gene expression would drop to the baseline level. Based on this prior knowledge, 7-day period of time point analysis of reporter gene expression was chosen for this study. It was expected that the highest gene expression to be detected would be at day 2 after instillation, as has been noted by several studies using GL67 as the gene carrier [31]. However, our results show that the highest gene expression was detected at day 1 after delivery (Figure 6(c)). Although a massive decline in gene expression was observed in the subsequent days, the RLU/mg values were all significantly higher than the controls.

Highest gene expression was observed at day 1 after delivery perhaps due to the CO_3_Ap pDNA release profile, which could be faster than PEI or GL67. Early release of pDNA would lead to faster protein translation, hence early detection of gene expression. The immediate release of the pDNA could be attributed to the chemical structure of CO_3_Ap, which is highly sensitive to the huge influx of H^+^ ion into the vesicle, thus making it swell and break the existing Van der Waals bonds between the CO_3_Ap matrix and the pDNA. The massive drop in gene expression seen after day 1 after delivery could probably be due to the pDNA being progressively exposed to the degradation by the lysosomal enzyme.

As mentioned, the gene expression from nonviral vectors generally reached an insignificant value by day 7 after delivery in mouse lungs [32]. However, the significantly high gene expression detected throughout the study time points signifies that the duration of gene expression mediated by the CO_3_Ap/pDNA was prolonged.

## 5. Conclusion

This study demonstrates that CO_3_Ap nanoparticles possess effective gene delivery property to the lung cell line and mouse airways. These findings suggest that CO_3_Ap exhibits attractive property well suited for gene delivery into the lung. Its noncytotoxic property and simplicity in formulation makes CO_3_Ap a simple and flexible gene carrier system to the lung cells. This study suggests CO_3_Ap/pDNA as an innovative and a novel approach for nonviral gene delivery and has future potential for lung gene therapy application.

## Figures and Tables

**Table 1:** Surface charges of various formulations of CO_3_Ap/pDNA complexes determined by Malvern charge analyzer. The charge densities became more negative with the increasing amount of CaCl_2_ in CO_3_Ap/pDNA complex formulations.

Sample	CO_3_Ap/pDNA complexes prepared with different volumes of CaCl_2_	Charge (mV)
1	4 *µ*L	0.25
2	5 *µ*L	−0.64
3	6 *µ*L	−12.06
4	9 *µ*L	−17.0
